# Good Mid-Term Clinical Outcomes and Low Arthroplasty Conversion Rates After Hip Arthroscopy with Labral Debridement Without Refixation or Reconstruction

**DOI:** 10.3390/jcm14093236

**Published:** 2025-05-07

**Authors:** Manuel Gahleitner, Daniel Hofer, Rainer Hochgatterer, Tobias Gotterbarm, Antonio Klasan

**Affiliations:** 1 Department of Orthopedics and Traumatology, Johannes Kepler University Linz, Kepler-University Hospital GmbH, 4020 Linz, Austria; manuel.gahleitner@kepleruniklinikum.at (M.G.);; 2 Orthopädie und Traumatologie, Klinik Diakonissen Linz, 4020 Linz, Austria; 3 AUVA Unfallkrankenhaus Steiermark, 8775 Kalwang, Austria

**Keywords:** FAI, hip arthroscopy, labral tears, cam impingement, pincer impingement, labral debridement

## Abstract

**Introduction**: The present study investigates the five-year outcomes of hip arthroscopy for cam or pincer-type femoroacetabular impingement (FAI) and associated labral tears in a defined patient population. **Methods**: Patients who underwent hip arthroscopy for cam or pincer-type arthroscopy femoroacetabular impingement (FAI) and labral tears at our hospital in the past five years were included. All patients who underwent revision—like a total hip arthroplasty (THA), a subsequent hip arthroscopy at another hospital, or had primary osseous diseases—were excluded. Patients were contacted via mail and asked to answer a clinical questionnaire called the “Hip Osteoarthritis Outcome Score” (HOOS) and to indicate whether there was a second surgery like a subsequent arthroscopy or THA. **Results**: There were 77 hip arthroscopies in 75 patients the last 5 years. A total of 29 patients responded. Those who did not respond were contacted via phone. All in all, we obtained the results of 49 patients (50 hips—29 right, 19 left, and 1 bilateral) who underwent hip arthroscopy over the past five years. The mean age at the time of operation was 41 years. Our results were as follows: 24 hips had an isolated labral tear, 49 hips a combined FAI pathology with cam and/or pincer-type impingement and labral tears, 3 patients had a posttraumatic FAI, and 1 patient suffered from hip chondromatosis, who was subsequently excluded; further, 22 patients (23 procedures) were lost to follow-up. HOOS contains various subscales; only the postoperative result of subscale 1 (symptoms) did not show a statistically significant improvement compared with the preoperative value. All other subscales showed a statistically significant improvement in comparison with the preoperative condition. Five patients (10.2%) still experienced symptoms, so we performed a total hip arthroplasty (THA) as a second surgical procedure. One patient was revised due to chondromatosis. One patient was revised at another center, and another was excluded because of chondromatosis. **Conclusions**: The five-year follow-up results of hip arthroscopy proved successful outcomes. Hip arthroscopy is an effective treatment for FAI in order to delay primary THA, regaining mobility and range of motion and reducing pain. Longer-term studies with a larger cohort are necessary.

## 1. Introduction

Femoroacetabular impingement (FAI) is increasingly being recognized as a significant cause of hip pain, restricted range of motion, and rapidly progressive osteoarthritis (OA) [1]. The gold standard for the treatment of FAI is hip arthroscopy, which yields reliable mid- to long-term results [2], and has become more widely adopted than surgical hip dislocation (SHD), as originally described by Ganz [3]. In patients with symptomatic FAI, an associated labral tear is found in up to 55% of cases [4]. When managing a labral tear, there are three surgical options: selective debridement, repair—if the tissue quality allows—or reconstruction [5]. While labral repair is considered the gold standard [6], it is technically more demanding than arthroscopic selective debridement, although less demanding than a reconstruction [5]. The learning curve for hip arthroscopy is known to be steep [7], with studies indicating limited improvement, even after more than 30 cases [8]. In the early phase of the arthroscopic learning curve, the additional technical steps required for labral refixation—such as anchor-specific portal placement, drilling, labral loading, and fixation—pose significant challenges, especially given that labral repair does not consistently result in superior outcomes for patients at this stage [9]. Nevertheless, recent consensus-based guidelines strongly advise against debridement in the majority of cases, particularly in younger patients [6,10]. The aim of this study was to evaluate clinical outcomes and conversion rate to total hip arthroplasty (THA) following the introduction of hip arthroscopy in a university hospital setting, during the initial implementation phase in which neither labral repair nor reconstruction were performed. This may be explained by the fact that, during the early phase of implementing the arthroscopic procedure and in the context of a low case volume, the outcomes of isolated labral resection are likely to be more reliable than those of labral repair [11]. Additionally, the average age of our patient population was higher compared with those in similar studies [12]. We hypothesized that clinical outcomes would be favorable, with a low rate of conversion to THA.

## 2. Materials and Methods

### 2.1. Patients

After a retrospective review of the prospectively collected data, consecutive patients who underwent hip arthroscopy for FAI and concomitant labral tears between 2016 and 2020 were included in the present study. A total of 77 hip arthroscopies were performed in the study period.

Patient age, time of surgery, time of conversion, and indication data were collected from the local electronic system as part of the operation−performance evaluation. The study received a local ethics board approval (1287/202).

### 2.2. Indications

**Figure 1:**
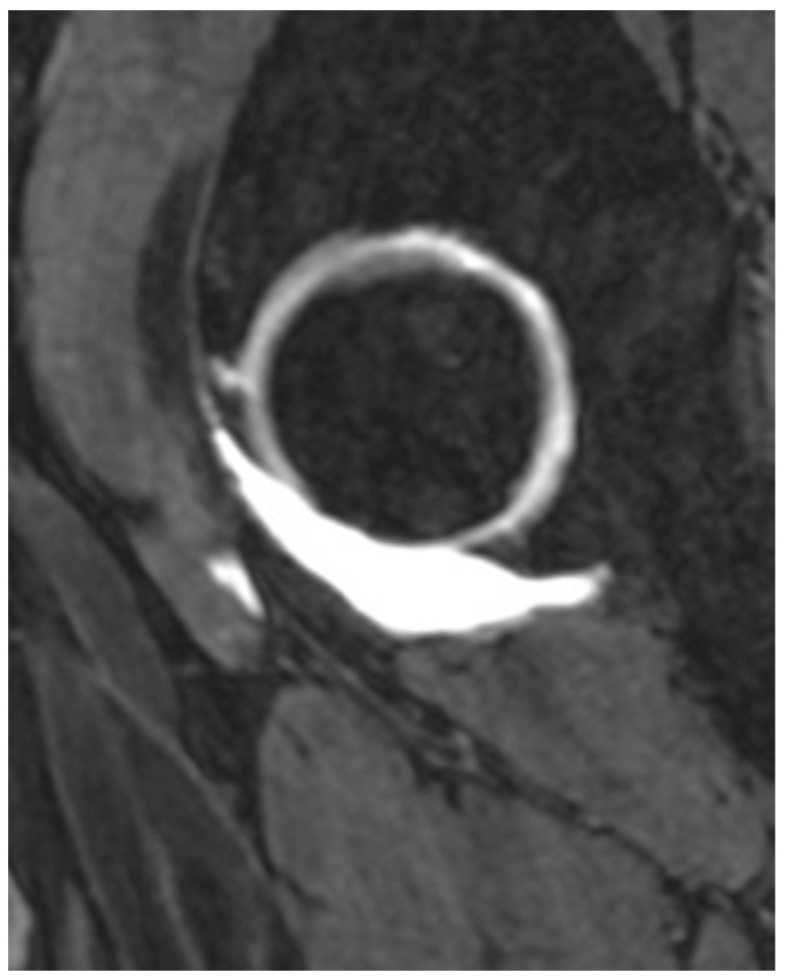
Arthro-MRI with labral tears.

All patients underwent preoperative anteroposterior and axial radiographs of the affected hip joints, as well as magnetic resonance imaging (MRI) using intra-articular contrast enhancement (Figure 1). During the clinical examination, gait, range of motion, muscle strength, points of tenderness, and signs of impingement or mechanical symptoms—such as snapping, catching, or locking—were systematically assessed.

All surgical procedures were performed by one of two arthroscopy surgeons. One surgeon initiated hip arthroscopy at the center, while the other had prior experience with approximately 30 hip arthroscopies before participating in this study. Therefore, both surgeons were at the beginning of the learning curve. The procedure was performed with the patient in a supine position on a standard traction table. After distraction, the anterolateral portal was established under fluoroscopy. Additional portals were established under direct visualization [13,14].

**Figure 2:**
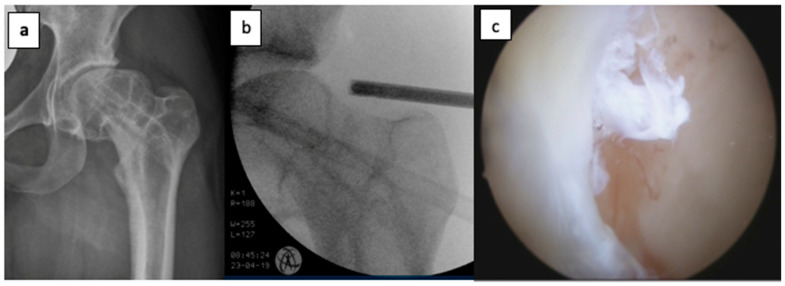
(**a**) Pre-operative X-ray of posttraumatic cam-impingement after slipped epiphysiolysis capitis femoris (SCFE); (**b**) intra-operative view of the same patient; (**c**) intra-operative labral tear.

Acetabuloplasty was performed to treat pincer-type impingement and femoroplasty was performed to treat cam-type impingement (Figure 2a,b). All labral tears were intra-operatively assessed. All tears were debrided until a stable labrum was achieved. This included tears at the labrum−capsule junction (Figure 2c). In 9 cases of cartilage damage, micro fracturing was performed.

Postoperatively, all patients were allowed to bear weight as tolerated, without restriction on rotation or flexion. This is an additional advantage of isolated labral resection. Full weight-bearing is permitted immediately postoperatively, allowing for a significantly faster and more effective return to daily activities compared with more complex labral refixation or reconstruction procedures.

In cases of micro fracturing, weight bearing was restricted to touch for 6 weeks. The first follow-up was performed 6–8 weeks after surgery, and yearly after that.

### 2.3. Follow-Up and Outcomes

The primary outcome was the occurrence of re-operations, including conversion to THA. The secondary outcome was patient-reported function, assessed preoperatively and at the latest follow-up using the Hip Osteoarthritis Outcome Score (HOOS) [15].

Exclusion criteria for statistical analysis included the presence of underlying osseous conditions (such as synovial chondromatosis), a second hip arthroscopy performed at an external institution, or patient refusal to participate in follow-up.

### 2.4. Statistics

SPSS version 26.0 (IBM, Armonk, NY, USA) was used for the statistical analysis. The Kolmogorov−Smirnov test was performed for testing for the normal distribution.

Normally distributed data are presented as mean (±SD) and non-normally distributed data are presented as median [interquartile range]. The Mann−Whitney U test was used to compare continuous variables. Correlations were calculated using the Pearson correlation coefficient. A formal power analysis was not performed as consecutive patients were included and significance was observed for the outcomes of interest. Additionally, we conducted a multiple linear regression to assess the statistical significance of the variables of age, sex, dysplasia, and alpha angle in relation to the HOOS score. A *p* value < 0.05 was considered statistically significant. To assess the potential impact of missing follow-up data, a sensitivity analysis was performed using best- and worst-case scenarios. In the best-case scenario, missing values were imputed using the group mean; in the worst case, no postoperative improvement was assumed for patients lost to follow-up.

## 3. Results

### 3.1. Demographic Data

Data evaluation revealed that 77 hip arthroscopies were performed in 75 patients between 2016 and 2020—33 on males (42.86%) and 44 on females (57.14%). A total of 34 procedures (44.16%) were performed on the left hip and 43 (55.84%) on the right. Two patients (2.67%) underwent staged bilateral hip arthroscopies. The mean age at the time of surgery was 42.92 years (SD ± 11.36). Surgical indications are summarized in Table 1. Approximately 19% of patients exhibited a mildly dysplastic acetabular configuration, as determined by Wiberg’s center-edge (CE) angle [16]. The mean alpha angle was 55.1 degrees (SD ± 7.04). Each MRI was independently assessed by three authors, and the mean of the three measurements was used for further analysis.

A complete follow-up of 49 patients was achieved. Two patients died of unrelated cause, but received no revision arthroscopy or arthroplasty according to their relatives. The mean time of follow-up was 35.46 months (SD ± 16.53). The arthroscopy due to hip chondromatosis sustained a relapse and resulted in subsequent hip arthroscopy two years after the first procedure. This patient was excluded from our cohort. Another patient underwent subsequent hip arthroscopy ex domo, with an indication being unknown. In total, 2 patients (3.8%) received a revision arthroscopy during the study period.

### 3.2. Conversion to THA

Five patients (9.8%) underwent conversion to THA following hip arthroscopy. The mean time from arthroscopy to THA conversion was 354 days (SD ± 344.9).

### 3.3. Postoperative Outcomes

A significant improvement in scores was achieved at the most recent follow-up (Table 2). Age and gender showed no significant correlation with the overall HOOS score or any of its subsections. Indication was not correlated with the preoperative or postoperative HOOS score. In the multivariate regression model, none of the included variables demonstrated a statistically significant association with the HOOS score. Age showed a negative but non-significant relationship with outcome (β = −0.43, *p* = 0.259), suggesting a slight trend toward lower scores with increasing age. Female gender was associated with higher HOOS scores (β = +8.72), although this was not statistically significant (*p* = 0.352). Similarly, the alpha angle showed a minimal positive association (β = +0.19, *p* = 0.756), and the presence of hip dysplasia was associated with slightly lower scores (β = −5.79, *p* = 0.610), but neither reached significance. Overall, the model suggests these patient characteristics do not substantially influence postoperative HOOS outcomes in this cohort (Table 3).

Using the method of calculating the minimal clinical important difference (MCID) for the HOOS score—defined as half a standard deviation—nearly all patients achieved a good result [17,18].

### 3.4. Impact of Loss to Follow-Up and Sensitivity Analysis

A total of 22 patients (29.3%) were lost to clinical follow-up and could not be included in the final outcome analysis. However, a review of the internal medical records confirmed that none of these patients underwent conversion to total hip arthroplasty (THA) within our hospital network, suggesting that no major surgical failure occurred in this subgroup. To estimate the potential effect of missing data on clinical outcomes, we conducted a simplified sensitivity analysis for the HOOS score results. For a best-case scenario, we assumed that all patients lost to follow-up experienced outcome improvements comparable to the cohort mean; in the worst-case scenario, we assumed no improvement or deterioration. While the best-case scenario did not meaningfully alter the results, the worst-case assumption reduced the statistical significance of the selected subscale improvements, although the overall direction of treatment effect remained favorable. This highlights the importance of follow-up completeness in observational outcome studies and is noted as a key limitation of our analysis.

## 4. Discussion

The most important finding of the present study is that hip arthroscopy—including labral debridement, femoroplasty, and acetabuloplasty—is an effective treatment for symptomatic FAI, even without labrum refixation or reconstruction. The present study also demonstrates a low conversion rate to THA after hip arthroscopy.

The outcomes of the study can be compared to the results of Sawyer et al. and Gupta et al., describing a conversion rate from 2% to 9%, respectively [12,19]. In a systematic review by Harris et al., the conversion rate was shown to be 2.9% [20]. In contrast with the aforementioned work by Sawyer et al., which reported mean age of 34.4 in hip arthroscopy, our population had a much higher mean age of 42.9. Thus, our conversation rate of nearly 10% might, on the one hand, be attributed to the increased age of our patients or the longer follow-up period of 35 months, compared to the 24 months reported in most of the mentioned studies [12,19,20]. Another reason may be the absence of labral refixation, which was not part of our surgical protocol. Compared with the study by Riff et al., the conversation rate for THA was also less than 10%. However, in their cohort, labral repair was performed considerably more often, whereas we never utilized this. This further confirms that a labral repair lowers the conversation rate to THA [21]. Our results describe the learning curve of two hip surgeons during their initial phase of hip arthroscopy. For this reason, more advanced techniques such as labral repair were not performed. The advantage of labral debridement is faster postoperative mobilization without restriction with respect to weight-bearing, in contrast to a four-stage rehabilitation program that takes 12 weeks for reconstructions [22]. Compared to labral repair or reconstruction, labral debridement has fewer complications, like perforation of the acetabular floor through a suture anchor or the subsequent development of scar tissue [23]. In the present study, a fairly high percentage, 19%, presented with dysplastic configuration of the acetabulum. This result might be slightly higher than the published data from Paliobeis et al. because we only referred to the CE-angle of Wiberg [16]. It might also reflect the early stage of implementation, where indications are not as strict. Byrd et al., however, reported a similar percentage of dysplasia compared to the present study [24]. A recent study by Murata et al. showed no difference in postoperative outcomes for patients with or without dysplasia [25]. In light of our findings, this might suggest that the indication for hip arthroscopy in patients with dysplasia could be provided more generously. Considering the work of Ayeni et al., labral tears associated with FAI can be treated using either labral debridement or labral repair. In five out of six studies, the postoperative outcomes of using labral repair were significantly better, but the direct postoperative situation and rehabilitation, including restriction of weightbearing, were much more restrictive [26]. Due to the difficult learning curve, we believe that only labral debridement should be considered for a center where this procedure is performed approximately 50 times per year, because this shows adequate and satisfactory results without an increased risk of complications, as shown in our study. This was also confirmed by Kelly et al. in 2005 [11]. If the case volume can be increased, we believe that labral repair should be considered in selected cases—particularly in young, athletic patients—and incorporated into the surgical repertoire. Our postoperative outcomes were evaluated using the HOOS score. Good clinical results were achieved, with an HOOS score of 8.4% at a mean follow-up of 35 months. The subscale of quality of life showed an increase of 16%. In comparison with the results of Ibrahim et al., we found better postoperative outcomes in every subscale [27]. Nguyen et al. showed similar results to ours in a two-year follow-up [28]. Similarly, Kucharik et al. found no difference between the two techniques at the final follow-up of the long-term study in hips that were not subsequently treated with total hip arthroplasty (THA). Notably, a higher conversion rate to THA was observed in the debridement group [29].

These results could support the legitimacy of hip arthroscopy for FAI and concomitant labral tears, even without labral repair.

The absence of data from 22 participants adversely affected the statistical analysis. However, patient records confirmed that none of these individuals underwent THA at any institution within our network, indicating no impact on the conversion rate. The effect of the missing data on the statistical outcomes of the HOOS score remains difficult to estimate.

Our multivariate regression analysis did not identify any statistically significant predictors of postoperative HOOS score among the evaluated variables—age, gender, alpha angle, and the presence of hip dysplasia. Although gender and dysplasia showed trends toward higher and lower HOOS scores, respectively, these effects did not reach statistical significance. The overall explanatory power of the model was low (R^2^ = 0.044), indicating that the demographic and radiographic parameters accounted for only a small fraction of the variability in postoperative outcomes. These findings suggest that commonly considered baseline characteristics, such as age and alpha angle, may not be sufficient on their own to predict patient-reported outcome measures following hip arthroscopy. This aligns with recent literature emphasizing the multifactorial nature of postoperative recovery, where intra-operative findings, surgical technique, rehabilitation protocols, and psychosocial factors may play a more dominant role in determining patient satisfaction and functional outcomes [30,31,32].

Furthermore, the lack of a significant association between dysplasia and HOOS score in our cohort could be attributed to the relatively small sample size or to the inclusion of only mild forms of dysplasia. Future studies with larger cohorts and more granular radiological assessments may be better suited to elucidate the impact of borderline or structural dysplasia on postoperative recovery.

In summary, while our model did not reveal significant predictors, the results highlight the complexity of outcome assessment in hip preservation surgery and underscore the need for more comprehensive, multidimensional models in future research.

### Limitations

There are a few limitations in the present study. About one third of the cohort was lost to follow-up, which had a significant influence on the overall result. Expanded learning curve data, such as time and state-trait analysis, were not thoroughly available and were, therefore, not analyzed. While this level of loss could potentially impact the statistical power and generalizability of our findings, we sought to mitigate its effect by verifying through institutional records that none of the patients lost to follow-up underwent THA within the affiliated hospital network. There have been multiple indications, and an analysis of the exact impact of each on the overall result would have reduced the power of the study.

## 5. Conclusions

In this cohort, hip arthroscopy for FAI and concomitant labral tears—performed without labral refixation or reconstruction—was associated with satisfactory short- to midterm clinical outcomes and a low conversion rate to arthroplasty. While these findings may support the use of technically simpler procedures in the early phase of adopting hip arthroscopy, they must be interpreted with caution due to the limited sample size and relatively high loss to follow-up. Further studies with larger cohorts and longer follow-up are needed to confirm these results and evaluate the long-term benefits of more advanced techniques.

## Figures and Tables

**Table 1:** Distribution of indications.

Indications	*n* (77)
Isolated labral tears	24 (28.57%)
Combined labral tears and cam/pincer impingement	49 (63.64%)
Posttraumatic	3 (3.9%)
Chondromatosis (excluded due to primary desease)	1 (1.3%)

**Table 2:** Outcomes of HOOS score, [IQR], *n* = 49.

	Preoperative Score	Postoperative Score	*p* Value
HOOS symptoms	13 [3]	14 [8]	*p* = 0.11
HOOS pain	25 [3]	29.5 [10]	*p* < 0.001
HOOS daily activity	48 [9]	56 [21]	*p* < 0.001
HOOS sport	10 [3]	11 [6]	*p* = 0.006
HOOS quality of life	9 [3]	11.5 [7]	*p* = 0.014
HOOS total (%)	67.6 [10.7]	76.0 [35.6]	*p* < 0.001

**Table 3:** Results of the multivariate regression model, without any statistically significant outcomes.

Predictor	Coefficient	95% CI	*p*-Value	Interpretation
Age	−0.43	[−1.19, 0.33]	0.259	No significant effect
Gender	+8.72	[−9.96, 27.40]	0.352	No significant difference (trend: higher scores in females)
Alpha angle	+0.19	[−1.01, 1.38]	0.756	No significant effect
Dysplasia	−5.79	[−28.49, 16.92]	0.610	No significant effect

## Data Availability

The ethical review committee supported this protocol with ethical and legal advice. Special thanks deserves the medical society of Upper-Austria for the generous financial support of our clinical research.

## Abbreviations

The following abbreviations are used in this manuscript:

FAI	Femuroacetabular impingement
HOOS	Hip Osteoarthritis Outcome Score
THA	Total hip arthroplasty
OA	Osteoarthritis
SHD	Surgical hip dislocation
MRI	Magnetic resonance imaging
SD	Standard deviation
IQR	Interquartile range
